# HPV Detection in Oropharyngeal Cancer: A Narrative Review of Diagnostic and Emerging Molecular Approaches †

**DOI:** 10.3390/diagnostics16071010

**Published:** 2026-03-27

**Authors:** Fernando López, Remco de Bree, M. P. Sreeram, Sandra Nuyts, Juan Pablo Rodrigo, Karthik N. Rao, Nabil F. Saba, Carol Bradford, Arlene Forastiere, Luiz P. Kowalski, Anna Luíza Damaceno Araújo, Carlos Suarez, Alfio Ferlito

**Affiliations:** 1 Department of Otolaryngology, Hospital Universitario Central de Asturias, Instituto de Investigación Sanitaria del Principado de Asturias, University of Oviedo, 33011 Oviedo, Spain; fernandolopezphd@gmail.com (F.L.); jprodrigo@uniovi.es (J.P.R.); 2 Instituto de Investigación Sanitaria del Principado de Asturias, University of Oviedo, 33011 Oviedo, Spain; csuareznieto@gmail.com; 3 Centro de Investigación Biomédica en Red de Cáncer (CIBERONC), 28029 Madrid, Spain; 4 Department of Head and Neck Surgical Oncology, University Medical Center Utrecht, 3584 Utrecht, The Netherlands; r.debree@umcutrecht.nl; 5 Department of Head and Neck Oncology, Sri Shankara Cancer Foundation, Bangalore 560004, India; sreeram.mp@sschrc.org (M.P.S.); karthiknagrao@gmail.com (K.N.R.); 6 Laboratory of Experimental Radiotherapy, Department of Oncology, KU Leuven, 3000 Leuven, Belgium; sandra.nuyts@uzleuven.be; 7 Department of Radiation Oncology, Leuven Cancer Institute, University Hospitals Leuven Institution, 3000 Leuven, Belgium; 8 Department of Hematology and Medical Oncology, Winship Cancer Institute, Emory University School of Medicine, Atlanta, GA 30322, USA; nfsaba@emory.edu; 9 Department of Otolaryngology-Head and Neck Surgery, The Ohio State University, Columbus, OH 43210, USA; carol.bradford@osumc.edu; 10 The Sidney Kimmel Comprehensive Cancer Center, Johns Hopkins, Baltimore, MD 21287, USA; af@jhmi.edu; 11 Head and Neck Surgery Department and LIM 28, University of São Paulo Medical School, São Paulo 05403-000, Brazil; lp_kowalski@uol.com.br; 12 Head and Neck Surgery and Otorhinolaryngology Department, A C Camargo Cancer Center, Sao Paulo 01509-900, Brazil; 13 Hospital Israelita Albert Einstein, Sao Paulo 05652-900, Brazil; anna_luizaf5ph@hotmail.com; 14 Coordinator of the International Head and Neck Scientific Group, 35100 Padua, Italy

**Keywords:** HPV, oropharyngeal squamous cell carcinoma, p16 immunohistochemistry, RNA ISH, ctHPV-DNA, liquid biopsy, diagnostics, artificial intelligence, de-escalation therapy

## Abstract

Human papillomavirus (HPV)-driven oropharyngeal squamous cell carcinoma (OPSCC) has emerged as a biologically distinct entity, typically affecting younger, non-smoking patients and showing improved survival compared to HPV-negative tumors. Accurate HPV status determination is essential for correct staging, prognostic assessment, and treatment de-escalation. Despite advances, substantial variability persists among diagnostic methods and clinical workflows. A narrative review of PubMed, Scopus, and Web of Science databases was conducted up to July 2025. Studies addressing HPV detection techniques in OPSCC—including p16^INK4a^ immunohistochemistry (IHC), HPV DNA and RNA assays, liquid biopsy approaches, and computational surrogates—were critically analyzed regarding diagnostic accuracy, clinical applicability, and emerging innovations. Tissue-based assays remain the diagnostic reference standard. p16 IHC provides high sensitivity but limited specificity and should be confirmed with nucleic acid-based methods such as DNA PCR, in situ hybridization (ISH), or E6/E7 mRNA detection. Combined or “orthogonal” testing minimizes discordance and refines risk stratification. Liquid biopsy detection of circulating HPV DNA using droplet digital PCR or next-generation sequencing has shown high sensitivity and specificity in cohorts of patients with HPV-associated OPSCC, supporting its potential role as a complementary biomarker for treatment monitoring and surveillance. However, circulating HPV DNA alone does not unequivocally identify the anatomic source of HPV DNA and should be interpreted together with clinical, radiologic, and tissue-based findings. Oral rinse and saliva assays show moderate diagnostic performance, while artificial intelligence-based radiomic and histopathologic models are emerging as complementary tools. Reliable HPV attribution in OPSCC requires a multimodal diagnostic strategy integrating p16 IHC, molecular confirmation, and ctHPV-DNA monitoring. Methodological standardization and prospective validation are essential to implement precision-guided, cost-effective workflows in routine clinical practice.

## 1. Introduction

Oropharyngeal squamous cell carcinoma (OPSCC) has seen a significant increase in incidence globally, particularly in high-resource and developed countries, with a projected rise of approximately 50% in incidence and mortality rates over the next 2 decades [1,2,3]. This epidemiological shift is largely attributed to the epidemic spread of high-risk human papillomavirus (HPV) infection, especially HPV-16, which is responsible for 40–80% of HPV-related OPSCC cases depending on the region [1,4,5]. Unlike OPSCC historically associated with tobacco and alcohol consumption, HPV-induced cancers represent a distinct clinical entity: patients are often younger, are frequently non-smokers, have less comorbidities, and display more favorable survival outcomes compared with their HPV-negative counterparts [2,6,7]. The recognition that HPV drives a distinct subset of OPSCC has led the American Joint Committee on Cancer (AJCC) to consider HPV-positive (HPV+) and HPV-negative (HPV−) disease as separate entities with different molecular profiles, tumor characteristics, staging and outcomes [6,8]. This distinct clinical and molecular profile highlights the critical importance of accurate HPV status determination for prognosis, inclusion in clinical trials on de-escalation approaches, and guiding treatment strategies in the near future [9].

Reliable and accurate diagnostic tools are essential for identifying truly HPV-driven OPSCC. Current approaches for HPV assessment in OPSCC usually involve a two-step strategy: initial detection with p16(INK4a) immunohistochemistry (IHC), followed by confirmation using HPV DNA Polymerase Chain Reaction (PCR) or E6/E7 mRNA in situ hybridization (ISH) [10]. While p16 IHC is often used as a surrogate marker for transcriptionally active HPV infection, multimodal testing that integrates two or more detection methods is recommended to ensure reliable identification and minimize false-positive or false-negative results [11]. Furthermore, the emergence of liquid biopsy techniques, such as the detection of cell-free HPV DNA (cfHPV-DNA) or circulating tumor tissue-modified viral HPV DNA (TTMV-HPV DNA) in blood and saliva, presents promising minimally invasive approaches for early diagnosis, treatment monitoring, and recurrence surveillance [4,12,13,14,15,16]. Despite advancements, the overall workflow from sample collection to result interpretation still requires further optimization and standardization to facilitate widespread clinical implementation.

Given the heterogeneity of available studies—covering diverse diagnostic methodologies, patient populations, and clinical applications—this review has adopted a narrative review methodology rather than a traditional systematic review. The aim is not to estimate a single pooled effect size, but rather to map the breadth of diagnostic approaches for HPV detection in OPSCC, summarize their strengths and limitations, and identify gaps that warrant further investigation. This approach allows for a comprehensive overview of existing evidence while acknowledging variability in study designs and outcomes.

This narrative review aims to provide a comprehensive synthesis of current diagnostic modalities for HPV detection in OPSCC, discuss their analytical performance and clinical implications, and explore emerging molecular and computational technologies that may reshape future diagnostic algorithms.

## 2. Literature Search and Selection Strategy

A comprehensive literature search was conducted in the PubMed/MEDLINE, Scopus, and Web of Science databases for studies published between January 2010 and July 2025, using combinations of the terms “human papillomavirus”, “HPV”, “oropharyngeal squamous cell carcinoma”, “p16 immunohistochemistry”, “HPV DNA”, “RNA ISH”, and “liquid biopsy”. Additional relevant articles were identified from reference lists of retrieved papers and review articles. Both original research articles and high-quality reviews published in English or Spanish were considered. Priority was given to studies providing clinical, diagnostic, or methodological insights relevant to HPV detection in OPSCC. Case reports, editorials, and non-peer-reviewed materials were excluded.

This review does not follow a systematic protocol but aims to synthesize and critically appraise the current evidence on HPV diagnostic methodologies, emphasizing clinical relevance, strengths, and emerging directions.

## 3. Results and Discussion

### 3.1. Epidemiology

Understanding the epidemiological dynamics of OPSCC is essential to contextualize the evolution of diagnostic strategies for HPV detection. The marked rise in HPV-related OPSCC over recent decades has redefined not only incidence patterns but also clinical behavior, patient demographics, and diagnostic needs.

HPV-positive OPSCC is among the most rapidly increasing cancers in high-income countries [17,18,19]. In the USA and UK, HPV currently accounts for approximately 71% and 52% of OPSCC cases, respectively, with meta-analyses showing a global prevalence near 44% [1]. However, considerable geographic variability has been reported, with attributable fractions ranging from less than 20% in Southern Europe to more than 60% in North America and Scandinavian countries: 63% in Denmark [20], 72% in Sweden [21], 77% in Norway [22], and up to 88% in the United States [5,20]. Subsite analysis reveals that the palatine tonsils and base of tongue are the predominant anatomical origins, reflecting the susceptibility of reticulated crypt epithelium [1]. Demographic differences are also evident: while OPSCC predominantly affects middle-aged men, some studies show higher HPV prevalence among women in specific regions [1]. Temporal trends further illustrate the disease’s evolution—incidence has surpassed that of cervical cancer among women in the US and UK [6], and data from Japan confirm a rising HPV-driven fraction of head and neck cancers over the past decade [17]. Behavioral factors, including oral sexual practices and lifetime number of partners, correlate with oral HPV prevalence, although recent population-based studies highlight the complexity and potential biases in quantifying such risks [19,23].

In summary, the epidemiological transition toward HPV-driven OPSCC highlights the need for precise and standardized diagnostic tools capable of differentiating virally induced from classical carcinogenesis. This shift underscores why HPV testing has become central to contemporary head and neck oncology.

### 3.2. Molecular Mechanisms

A clear comprehension of HPV-driven molecular oncogenesis provides the biological foundation for selecting and interpreting diagnostic tests. The viral life cycle, integration events, and gene expression patterns directly influence the molecular signatures that current assays attempt to detect.

The molecular mechanisms underlying HPV oncogenesis represent one of the most elegant yet devastating examples of viral subversion of host cellular control. High-risk HPV types—particularly HPV16 and HPV18—encode the oncoproteins E5, E6, and E7, which cooperatively disrupt key tumor suppressor and immune regulatory pathways, leading to uncontrolled proliferation, genomic instability, and malignant transformation.

The E6 protein mediates the degradation of the p53 tumor suppressor through its interaction with the E6-associated protein (E6AP), thereby impairing DNA damage response, suppressing apoptosis, and promoting telomerase activation via hTERT upregulation. In parallel, the E7 oncoprotein binds to the retinoblastoma (pRb) protein, promoting its proteasomal degradation and subsequent release of E2F transcription factors, which drive premature S-phase entry and dysregulated cell cycle progression. The E5 protein contributes to immune evasion by downregulating major histocompatibility complex (MHC) class I molecules, allowing infected cells to escape cytotoxic T-cell surveillance. Collectively, these alterations generate a cellular environment characterized by proliferative immortality, apoptosis resistance, chromosomal instability, and immune escape—hallmarks of HPV-mediated carcinogenesis [1,2,5,12,24].

In the oropharynx, persistent HPV infection and immune evasion within the tonsillar crypt microenvironment facilitate the clonal expansion of transformed epithelial cells, constituting critical steps in the pathogenesis of HPV-related OPSCC [5]. Molecular profiling has revealed that HPV-positive OPSCCs display distinct transcriptional and mutational landscapes compared with HPV-negative counterparts, including fewer TP53 mutations and unique immune signatures. These differences not only explain the improved prognosis of HPV-associated tumors but also provide a biological rationale for biomarker-driven therapeutic de-escalation and immunotherapy strategies [2,12].

HPV16 remains the predominant genotype, accounting for up to 80% of HPV-positive OPSCC cases, followed by HPV18, HPV33, HPV35, and HPV58 [1,5]. Nonetheless, other high-risk genotypes also contribute significantly to carcinogenesis. Approximately 6% of HPV-positive oropharyngeal tumors are attributable to non-HPV16 types—mainly 18, 33, 35, and 39. In contrast, nasopharyngeal and oral cavity tumors exhibit greater genotype heterogeneity, with over half of HPV-positive cases linked to non-16 genotypes such as HPV18, 31, 35, 39, 58, or 66. This variability underscores the importance of incorporating broad high-risk HPV genotyping into diagnostic algorithms, as the detection of non-16 subtypes should not be considered incidental [25]. From a diagnostic perspective, the predominance of HPV16 has influenced the development and validation of many detection assays. Most tissue-based molecular techniques, including multiplex PCR and MassArray-based genotyping platforms, are capable of identifying a broad spectrum of high-risk HPV genotypes and therefore show comparable analytical performance across HPV16 and non-HPV16 types. In contrast, several emerging diagnostic strategies have been primarily optimized using HPV16-driven tumors. For example, many circulating tumor HPV DNA assays target HPV16-specific sequences, although newer platforms are expanding coverage to additional high-risk genotypes such as HPV18, 31, 33, and 35. Similarly, computational models trained to predict HPV status from imaging or histopathology often rely on datasets dominated by HPV16-positive tumors, and their performance across less common genotypes remains insufficiently characterized. Future studies including more diverse genotype distributions will therefore be essential to determine whether these emerging diagnostic approaches maintain comparable sensitivity across the full spectrum of oncogenic HPV types.

Taken together, these molecular insights reveal that HPV-driven OPSCC is not a mere viral presence but a transcriptionally active and biologically distinct entity. Understanding these mechanisms not only clarifies the molecular basis of HPV-mediated oncogenesis but also provides the rationale for validating biomarkers and developing diagnostic assays that reflect true viral oncogenic activity rather than incidental infection. The following sections examine how these molecular insights have guided the evolution of current and emerging HPV detection methods.

### 3.3. Methods of HPV Detection in Oropharyngeal Carcinoma

The diagnostic landscape for HPV detection in OPSCC has evolved dramatically, moving from surrogate histopathological markers toward integrated molecular and liquid-biopsy-based strategies. The choice of method directly affects clinical staging, prognostic stratification, and the feasibility of treatment de-intensification. A summary of the key studies evaluating HPV detection methods, including tissue-based, liquid biopsy, and computational approaches, is provided in Table 1.

Determining whether an OPSCC is HPV-driven has direct consequences for staging, prognosis, patient counseling, and—critically—the design/eligibility of de-escalation trials. The testing landscape has evolved from morphologic surrogates toward integrated molecular and liquid-biopsy strategies. We review tissue-based assays (surrogates and direct detection), combined algorithms, non-invasive approaches (oral samples and blood-based ctHPV-DNA), and practical considerations that affect real-world performance.

#### 3.3.1. Tissue-Based Testing

The identification of HPV-positive OPSCC has become central to contemporary head and neck oncology. These tumors constitute a biologically distinct entity with specific molecular characteristics and a significantly more favorable prognosis than HPV-negative disease. Accurate assessment of HPV status is therefore critical for prognostic evaluation and therapeutic decision-making [26].

Tissue-based assays remain the foundation of HPV detection in OPSCC, as they provide direct access to tumor material and enable histopathological correlation. Among these, p16 immunohistochemistry (IHC) has emerged as the most widely implemented surrogate marker due to its technical simplicity, reproducibility, and high sensitivity. In situ hybridization (ISH), particularly RNA ISH targeting E6/E7 transcripts, offers higher specificity by demonstrating transcriptionally active HPV within tumor cells and is frequently used as a confirmatory method in diagnostically equivocal cases or research settings. Consequently, tissue-based testing continues to represent the diagnostic reference standard against which novel and emerging detection methods must be validated to ensure interinstitutional comparability and clinical trial consistency.

**Table 1:** Summary of key studies on HPV detection in oropharyngeal squamous cell carcinoma (OPSCC): methodologies, settings, and main findings.

Reference	Study Design	Population/Setting	Detection Methods	Main Findings
**Tissue-Based Testing**				
**Walline et al. [25]**	Comparative methods study	Oropharynx, nasopharynx, oral cavity (n = 338)	p16 IHC, DNA ISH, PCR-MassArray; PGMY-PCR + sequencing for discordance	PCR-MassArray: 99.5% SN/100% SP; p16: 94.2%/85.5%; ISH: 82.9%/81.0%; ~86% hrHPV+ in OPSCC, HPV16 predominant.
**Shinn et al. [27]**	Retrospective multicenter cohort	OPSCC	p16 IHC (≥70%), HPV E6/E7 mRNA RT-qPCR	p16/HPV mRNA discordance ~5–10%; p16+/HPV− group shows intermediate prognosis vs. concordant groups.
**Mehanna et al. [28]**	Multinational individual patient data	OPSCC (13 cohorts; n = 7654)	p16 IHC + HPV testing (DNA/RNA)	p16/HPV discordance 10.9% among p16+; 5-year OS: p16+/HPV+ 81%, p16−/HPV− 40%, p16+/HPV− 55%, p16−/HPV+ 53%.
**Bhardwaj et al. [29]**	Consecutive clinical series	OPSCC, sinonasal, CUP (n = 746)	p16 IHC; HPV DNA PCR; E6/E7 RNA ISH; genotyping (MassArray) on discordant cases	p16 vs. PCR concordance 95.6%; p16 sens 99.8%, spec 92.1%; false p16+ linked to non-16 genotypes.
**Ferrandino et al. [30]**	Surveillance cohort	HPV+ OPSCC on routine surveillance with TTMV-HPV DNA (NavDx)	Serial TTMV-HPV DNA testing; clinical correlation	Very high NPV for recurrence exclusion; undetectable TTMV-HPV DNA strongly associated with absence of disease on follow-up.
**Liquid biopsy (ctHPV DNA)**				
**Nguyen et al. [31]**	Prospective liquid biopsy comparison	28 OPCSCC (locally advanced n = 22; metastatic n = 6)	cf-DNA/RNA vs. EV-DNA/RNA ddPCR for HPV; longitudinal sampling	cf-DNA more sensitive than EV-DNA (91% vs. 42%) and cf-RNA > EV-RNA (83% vs. 50%); clearance post-curative therapy in LA disease.
**Veyer et al. [32]**	Prospective biomarker cohort	OPSCC p16+/HPV16+; baseline plasma before therapy (n = 66)	ddPCR quantification of HPV16 ctDNA	Baseline ctDNA detected in 71%; correlated with stage; early kinetics aligned with outcomes.
**Chera et al. [33]**	Prospective multicenter biomarker trial	OPSCC p16+ M0; definitive CRT (n = 103)	Plasma ctHPV DNA (16/18/31/33/35) by dPCR; baseline and weekly during CRT	Diagnosis: 89% SN/97% SP; >95% clearance by day 28 predicted improved locoregional control; supports de-escalation concepts.
**Tanaka et al. [34]**	Prospective cohort with post-RT assessment	HPV16-related HNSCC treated with RT ± CT (n = 35 HPV16 HNSCC)	Post-RT plasma ctHPV16 DNA (ddPCR) + PET-CT metabolic response	Post-RT ctHPV16 DNA PLR 100% vs. PET-CT PLR 50%; similar NLR (~90%); ctHPV16 DNA complements PET-CT for salvage decisions.
**Mena et al. [35]**	International multicenter study (ICO)	OCC/OPC/larynx HPV-DNA+	Algorithm: HPV DNA → p16 IHC; reference E6*I mRNA	Concordance p16 vs. mRNA: OPC 82.1%, OCC 79.5%, larynx 56.9%; better with p16 cutoff > 50%; genotype ≠16 shows different patterns.
**Cao et al. [36]**	Prospective cohort	OPSCC p16+ stage III (n = 34)	Serial ctDNA + MRI/PET during CRT	Early ctDNA kinetics (week 2) predicted tumor control; correlated with imaging biomarkers.
**Rosenberg et al. [37]**	Prospective biomarker trial with NACT and adaptive de-escalation	46 locally advanced HPV+ OPSCC	Serial ctHPV DNA by NGS	≥95% ctDNA clearance after 1 NACT cycle predicted response; post-treatment ctDNA predicted recurrence (PPV/NPV 100%), lead time up to 25 months.
**Regan et al. [38]**	Prospective phase II (CRT + nivolumab)	26 locally advanced p16+ OPSCC	Serial ctDNA; functional MRI/PET	Nivolumab did not improve PFS (65% at 2 y) and increased toxicity; ctDNA kinetics not associated with survival; functional imaging useful.
**Oral specimens**				
**Fakhry et al. [39]**	Prospective multicenter cohort (n ≈ 396)	Oral/oropharyngeal HNSCC	Serial oral rinse qPCR (multigenotype)	For HPV16+ tumors: oral rinse 81% SN, 100% SP; post-treatment persistence associated with worse OS and higher recurrence.
**Tanaka et al. [40]**	Prospective diagnostic study	74 OPC and 8 CUP	Oral HPV DNA (GENOSEARCH HPV31), oral HPV mRNA (Aptima), plasma ctHPV16 DNA (ddPCR)	For HPV16 OPC: oral DNA 82/100%, oral mRNA 85/94%, ctHPV16 DNA 93/97%; ctDNA correlated with tumor burden/genomics.
**Hillier et al. [41]**	Prospective cohort	Benign tonsillectomy population (n ≈ 945)	Oral rinse and brush; SPF10-LiPA25; HPV serology	Oral rinse detected more cases (hrHPV ~4%) but missed ~73% positives found at other sites; combining sampling improves detection.
**Radiomics**				
**Bos et al. [42]**	External validation of MR-based radiomic model for LRC	External OPSCC cohort (n = 157); pre-RT MRI	Validation of prior MR-radiomic model	AUC 0.64 externally; improved with matched HPV (0.68), 4 mm slices (0.67), or quantile harmonization (0.66).
**Leijenaar et al. [43]**	Multicenter radiomics development/validation	Four independent OPSCC cohorts (total n ≈ 778)	Pre-treatment CT radiomics (902 features) + LASSO; artifact-aware training/validation	AUC ~0.70–0.80 across validations; radiomics separated KM curves similar to p16; proof-of-concept that CT radiomics predicts HPV (p16).
**Ahmadian et al. [44]**	Radiogenomics methods (post-processing impact)	OPSCC MRI: train 91, test 62, external 157	CE-T1 MRI radiomics; harmonization; unstable/correlated feature removal	Without post-proc: AUC 0.79 (test) → 0.52 (external); with post-proc: AUC 0.76 (test) and 0.73 (external).
**Digital Pathology and Artificial Intelligence**				
**Klein et al. [45]**	Multicenter retrospective; development/validation of DL classifier	OPSCC (Giessen n = 163; Cologne n = 110) + TCGA HNSCC (n = 329)	Deep learning on H&E WSI (U-Net + DenseNet) to derive HPV prediction score (HPV-ps); p16 IHC; HPV DNA PCR	HPV-ps AUC 0.80 in two independent OPSCC cohorts; favorable prognosis (HR 0.44–0.69). Combining HPV-ps with p16 improved stratification (HR 0.06–0.30).
**Wang et al. [46]**	Multicenter retrospective; develop/validate DL Digital-HPV Score	TCGA-HNSC (n = 412); Sheffield-OPSCC (n = 69); FFPE H&E	Multiple-instance learning + triplet-ranking on WSI H&E; validated vs. p16 IHC and E6/E7 ISH	External AUC 0.84–0.92; stratified OS/DSS comparable to molecular tests; HPV+ associated with higher B/T-cell and lower macrophage infiltration.
**Song et al. [47]**	Multicenter retrospective cohort (SMuRF multimodal AI)	277 HPV+ OPSCC with paired CT and WSI	Swin-Transformer multimodal (primary tumor and nodes on CT + WSI); cross-attention	Predicted DFS (C-index 0.79–0.81) and grade (AUC 0.74–0.75); HR 17 (*p* < 0.0001) independent; outperformed unimodal models.
**Systematic reviews/meta-analyses/clinical updates**				
**Mehanna et al. [48]**	Narrative clinical update	OPSCC; clinical synthesis	p16; DNA/RNA PCR/ISH; dual-testing; ctDNA	RNA RT-PCR is most accurate; dual-testing identifies ~9.2% discordant with worse prognosis; improves TNM/prognostic accuracy.
**Jakobsen et al. [49]**	Systematic review and meta-analysis	OPSCC (27 studies; n = 5488)	p16 IHC, DNA ISH/PCR, mRNA RT-PCR; ctDNA; saliva/oral rinse	Tissue: 81–93% SN, 81–95% SP; blood: 81%/95%; oral: 77%/74%. Blood shows higher clinical promise for diagnosis.
**Paolini et al. [15]**	Systematic review and meta-analysis	OPSCC with diagnostic ctHPV DNA (13 studies; n = 998)	Plasma ctHPV DNA by ddPCR	Diagnosis: 90% SN, 94% SP; PLR 12.6, NLR− 0.05; supports clinical utility of ctDNA.
**Campo et al. [4]**	Systematic review and meta-analysis	HPV+ OPSCC (12 studies; n = 1311)	ctHPV DNA and TTMV-HPV DNA (ddPCR) for surveillance	Recurrence: 86% SN, 96% SP; AUC 0.81; promising for early surveillance (needs standardization).
**Poljak et al. [16]**	Mini-review (laboratory update)	Urine, blood, oral sampling	ddPCR, qPCR, NGS; first-void urine devices; ctDNA; oral rinses	Blood ctDNA promising for diagnosis/surveillance; oral rinses moderate sensitivity; first-void urine useful (female screening).
**Lu et al. [50]**	Systematic review and meta-analysis	Global OPSCC (134 studies; 12,139 cases)	HPV DNA PCR; p16 IHC; regional/genotype analyses	Overall prevalence 48.1% (N. America 72.6%); HPV16 40.2%; high p16/HPV concordance; regional variability.
**Khan et al. [14]**	Narrative update	Global HNSCC/OPSCC	p16 IHC, DNA/RNA ISH, DNA PCR, RNA RT-PCR, RNA ISH, ctDNA, E6 antibodies	Recommend p16 as initial screen followed by confirmatory HPV test; RNA ISH and ctDNA emerging with high clinical value.

**AUC**: area under the curve; **ctHPV**: circulating tumor HPV DNA; **CRT**: chemo-radiotherapy; **CT**: computed tomography; **ddPCR**: droplet digital PCR; **DFS**: disease-free survival; **hrHPV**: high-risk HPV serotypes; **HPV**: human papillomavirus; **IHC**: immunohistochemistry; **ISH**: in situ hybridization; **LCR**: loco-regional control; **NGS**: next-generation sequencing; **MRI**: magnetic resonance imaging; **NLR**: negative likelihood ratio; **OPSCC**: oropharyngeal squamous cell carcinoma; **OS**: overall survival; **PCR**: polymerase chain reaction; **PLR**: positive likelihood ratio; **PGMY-PCR**: polymerase chain reaction using PGMY primers; **qPCR**: quantitative PCR; **RT**: radiotherapy; **SN**: sensitivity; **SP**: specificity; **TTMV**: tumor tissue-modified viral; **WSI**: whole-slide images.

##### **p16 IHC: Strengths, Limits, and Cut-Offs** 

Among diagnostic modalities, p16 IHC is the most widely adopted method for determining HPV association in OPSCC. Overexpression of p16^INK4a^ reflects E7-mediated inactivation of the retinoblastoma (pRb) pathway, leading to compensatory p16 accumulation [51,52]. This marker provides an inexpensive, reproducible, and formalin-fixed paraffin-embedded (FFPE)-compatible surrogate of transcriptionally active HPV infection, enabling broad clinical applicability [53]. International guidelines recommend reporting p16 positivity when ≥70% of tumor cells display strong nuclear and cytoplasmic staining—a criterion incorporated into AJCC TNM-8 and the upcoming TNM-9 staging systems for OPSCC [8,54].

Clinically, p16 positivity correlates strongly with improved prognosis. Large series have shown 5-year overall survival (OS) rates of approximately 74% in p16-positive versus 44% in p16-negative OPSCC [7]. Different cut-offs (50%, 70%, 75%) have been evaluated, but a ≥70% threshold offers optimal prognostic discrimination and reproducibility across laboratories [54].

Assay performance can vary depending on the antibody clone. The E6H4 clone provides greater staining intensity, reduced interobserver variability, and superior prognostic stratification compared with JC8 and G175-405 [53]. When non-E6H4 clones are used, a lower 50% cut-off may improve discrimination.

Despite its utility, p16 IHC remains an indirect marker of HPV-driven oncogenesis. False positives (p16-positive/HPV-negative) may occur due to HPV-independent RB1 pathway inactivation [55,56,57], while false negatives can arise when HPV DNA or mRNA is present without p16 overexpression [58,59,60]. Meta-analyses show that among HPV-positive OPSCCs confirmed by nucleic acid-based methods, 8–13% are p16 negative [50], consistent with large cohort data reporting 3–5% p16-negative but HPV mRNA-positive cases [27,29]. False-positive p16 immunostaining (p16-positive/HPV-negative) reflects HPV-independent alterations in the RB1 pathway or CDKN2A gene, which lead to aberrant p16 overexpression despite the absence of transcriptionally active HPV. False-negative p16 results can occur in transcriptionally active HPV-positive OPSC due to low or heterogeneous p16 expression, assay variability, or biological factors affecting pRb pathway deregulation. On fine-needle aspiration (FNA) specimens of nodal metastases, cytologic processing variability may necessitate lower thresholds to maintain sensitivity.

Overall, pooled analyses across 23 studies have demonstrated that p16 IHC achieves a sensitivity of approximately 94% and specificity of 83% for detecting HPV-related OPSCC, with specificity improving to 96% when combined with HPV DNA PCR [61]. Thus, while p16 IHC provides a robust and practical first-line surrogate for HPV-driven oncogenesis, optimal diagnostic accuracy is achieved through integration with nucleic acid-based confirmatory assays.

##### **Direct Viral Detection in Tissue: DNA PCR, DNA ISH, and RNA Assays** 

As with antibody clone selection in immunohistochemistry, the performance of HPV DNA and RNA detection assays is strongly influenced by the design of primers and probes. Differences in targeted genomic regions—most commonly the L1 region versus the E6/E7 oncogenes—can significantly impact analytical sensitivity and specificity. PCR assays targeting L1 may be affected by viral integration events that disrupt this region, potentially leading to false-negative results, whereas assays targeting E6/E7 are generally considered more reliable indicators of transcriptionally active infection. Similarly, in situ hybridization techniques vary according to probe design, including signal amplification strategies and the range of HPV genotypes covered. Multiplex PCR and MassArray-based platforms allow simultaneous detection of multiple high-risk HPV genotypes, although their performance also depends on primer design and coverage of relevant viral subtypes. Therefore, careful selection and validation of primers and probes are essential for accurate HPV detection, although standardization across platforms remains limited.

The landmark study by Gillison et al. [62] established the etiological and prognostic significance of HPV in head and neck cancers. In a cohort of 253 patients with head and neck squamous cell carcinoma (HNSCC) treated between 1987 and 1998, HPV was detected in 25% of cases, with HPV16 accounting for 90% of positive tumors. The highest prevalence was observed in OPSCC (odds ratio = 6.2; 95% CI, 3.1–12.1). Detection methods included PCR, Southern blot hybridization, and ISH, confirming nuclear localization of HPV16 DNA. Importantly, HPV-positive tumors demonstrated significantly improved survival, establishing HPV-positive OPSCC as a distinct clinical and molecular entity.

HPV DNA PCR (type-specific or consensus) is highly sensitive but cannot distinguish latent/“passenger” infection from true viral oncogenic activity. Tumors that are HPV DNA-positive but p16 negative tend to behave clinically like HPV-negative cancers, underscoring the need for complementary testing [63]. DNA in situ hybridization (ISH) trades some sensitivity for morphologic localization of viral genomes, improving clinical specificity. E6/E7 RNA detection—by RT-PCR or RNA ISH—directly demonstrates oncogene transcription and is widely regarded as the functional gold standard despite greater technical complexity and costs, because it provides the most specific evidence of biologically active HPV infection [64].

A comprehensive meta-analysis in OPSCC found high pooled diagnostic performance across tissue techniques (although reference methods varied between studies), for example: RNA ISH sensitivity 93.4% and specificity 92.4%; DNA ISH 86.3% and 95.3%; DNA PCR 83.5% and 89.1% [49]. In a cohort of 338 head and neck cancer specimens, comparative assay evaluation demonstrated that PCR-MassArray achieved 99.5% sensitivity and 100% specificity for high-risk HPV detection in FFPE tissue, outperforming p16 immunohistochemistry (94.2% and 85.5%, respectively) and in situ hybridization (82.9% and 81%). Beyond accuracy, the technique requires minimal DNA input (5 ng) and enables simultaneous identification of multiple HPV genotypes. The study included tumors from the oropharynx, nasopharynx, and oral cavity, and the superior analytical performance of PCR-MassArray was maintained across these anatomical subsites. However, these results were obtained in a single-institutional cohort and therefore may not fully reflect variability across different geographic populations or HPV genotype distributions. Moreover, despite its high analytical performance, PCR-MassArray is currently implemented mainly in specialized or research laboratories and has not yet been widely adopted in routine clinical diagnostic workflows [25].

The quality of fixation, deparaffination, and age of FFPE samples degrade nucleic acids and can reduce the sensitivity of PCR and RNA ISH. Although RNA ISH is increasingly proposed as a single solution when available (high accuracy, in situ localization), it is not widely used, and DNA-based methods remain fundamental in many laboratories.

Overall, tissue-based assays provide robust diagnostic accuracy when used appropriately, but no single test is infallible. The continued reliance on p16 IHC demands careful contextual interpretation, and integration with direct viral detection remains essential for reliable HPV attribution, particularly in clinical trials and de-escalation protocols. In clinical reality, tissue-based testing remains the diagnostic gold standard against which emerging assays must be benchmarked, ensuring consistency across institutions and trials.

#### 3.3.2. Combined Testing and the Problem of Discordance

Because neither p16 expression nor viral DNA detection alone can fully capture transcriptional activity, combined or “orthogonal” testing approaches have become the gold standard for reliable HPV classification.

##### **Why Dual-Testing Matters** 

Because p16 is exquisitely sensitive yet not perfectly specific, and DNA positivity may not equate to transcriptional activity, combining p16 IHC with a direct HPV test (DNA or RNA) yields the most reliable classification. The large HNCIG-EPIC-OPC individual-patient analysis and subsequent syntheses emphasize that discordant phenotypes (p16+/HPV− or p16−/HPV+) are not rare (~9–10%): 10.9% of all p16+ OPSCC was HPV− and 7.5% of all p16- OPSCC was HPV+. Moreover, these intermediate outcomes carry prognoses significantly worse than double-positive and better than double-negative, undermining de-escalation if p16 alone is used [48].

In the HNCIG-EPIC-OPC study, the 5-year OS was 81% for p16+/HPV+, 55% for p16+/HPV−, 53% for p16−/HPV+, and 40% for p16−/HPV−; smoking further worsened outcomes among p16+/HPV− cases. These findings support routine dual-testing where results inform trials, counseling, or anticipated treatment intensity [28].

In the large comparative series by Walline et al. [25], discordant cases between detection methods were systematically evaluated. Among 25 tumors that were PCR-MassArray-negative but positive by p16 or ISH, repeat testing with consensus L1 PCR and sequencing confirmed negativity in 24 cases, with only one tumor ultimately identified as HPV16-positive. These results suggest that most discordances represent false positives from p16 or ISH, reinforcing the value of combining PCR-MassArray with p16 IHC as a robust strategy for accurate HPV classification.

##### **Geography, Subsite, and Biology** 

Discordance varies by region (higher where HPV-attributable fraction is lower, e.g., Southern Europe) and by oropharyngeal subsite, with non-palatine/lingual-tonsil primaries showing higher p16+/HPV− rates—arguing for confirmatory testing particularly in these regions and subsites. Accordingly, large investigations recommend dual-testing as standard in de-escalation trials and suggest regional tailoring of clinical practice depending on background prevalence/discordance [28].

Beyond OPSCC, an international study comparing p16 vs. E6*I mRNA concordance across head and neck sites showed substantial triple-positivity (HPV DNA/p16/E6*I mRNA) in the oropharynx, but markedly lower in the oral cavity and larynx—underscoring that p16 (alone or combined) is not reliable for diagnosing HPV-driven disease outside the oropharynx [35].

In short, dual-testing strategies reduce diagnostic ambiguity and mitigate the prognostic uncertainty associated with discordant phenotypes. Future guidelines should encourage confirmatory molecular testing—especially in low-prevalence settings—before therapeutic de-escalation decisions are made. Ultimately, the integration of p16 IHC and molecular detection represents not just a technical optimization but a paradigm of precision pathology—balancing feasibility with biological validity.

Current clinical guidelines reflect the central role of HPV status determination in OPSCC management. The AJCC 9th edition staging system incorporates p16 immunohistochemistry as a surrogate marker of HPV-associated disease, and p16 positivity is currently used for clinical staging in routine practice [8]. Similarly, the National Comprehensive Cancer Network (NCCN) guidelines recommend p16 testing for all newly diagnosed OPSCCs as part of the diagnostic work-up. However, increasing evidence has highlighted the limitations of relying solely on p16 expression, particularly due to the recognized proportion of p16-positive/HPV-negative tumors and the prognostic heterogeneity of discordant cases. The studies reviewed here support the growing consensus that combined or “orthogonal” testing strategies—including p16 immunohistochemistry with confirmatory HPV DNA or RNA detection—may provide more accurate classification, especially in clinical trials evaluating treatment de-escalation. Emerging diagnostic approaches such as circulating tumor HPV DNA and computational biomarkers are not yet incorporated into current guidelines but may inform future updates once prospective validation and methodological standardization are achieved.

#### 3.3.3. Non-Invasive Approaches

Efforts to develop non-invasive HPV detection methods have been driven by the need for accessible, patient-friendly, and repeatable sampling. Oral rinse and saliva testing have attracted attention for their potential in population surveillance and longitudinal follow-up.

##### **Oral Specimens (Rinse, Swab, Saliva)** 

From a population perspective, oral sampling is attractive for epidemiology of HPV infections, vaccine impact, and as an adjunct where tissue is difficult to obtain. However, at diagnosis, performance is modest. A meta-analysis cited pooled sensitivity ~77% and specificity ~74% for oral samples in HPV+ OPC, with high inter-study variability. Other summaries report specificity ~92% but lower sensitivity vs. tissue and note prognostic associations with higher oral HPV16 load at diagnosis and post-treatment persistence [14,35].

The Oromouth cohort provides a contemporary and methodologically rigorous dataset. This study enrolled 945 participants, aged 0–65 years (63% female), undergoing tonsillectomy for non-malignant indications, without OPSCC diagnoses. Samples from oral rinse, pharyngeal wall, tongue base, tonsil tissue and blood were collected. Oral rinse sampling achieved the highest detection rate among oral sites; however, it still failed to identify approximately 73% of HPV infections detected at other oropharyngeal subsites, particularly the base of tongue and tonsil. The addition of serology did not materially enhance detection performance. The authors thus concluded that oral rinse is a useful epidemiologic tool and a pragmatic adjunct, but its limitations must be recognized in screening/diagnosis and in vaccine-impact studies that use oral rinse as a primary endpoint [41].

This technique of oral rinse may serve as a useful adjunct when tissue biopsy is delayed or contraindicated. In addition, it could contribute to tertiary prevention, as persistence of oral HPV16 DNA following treatment has been associated with an increased risk of early recurrence [39].

As an alternative to oral rinse, if oral lesions are visible, the mucosal surface could be sampled using a swab or brush. Saliva is not considered a robust specimen for HPV testing, nor are specimens obtained by swabbing or brushing the tonsils or the oropharynx without visible lesions. The moderate sensitivity of these alternatives currently limits their use as diagnostic tools [16].

Despite their convenience, oral-based assays still lack the sensitivity required for diagnostic replacement of tissue testing. Their greatest promise lies in epidemiologic surveillance and post-treatment monitoring, not as stand-alone diagnostic tools for OPSCC. Future research should focus on refining assay sensitivity and standardizing sampling to enable oral-based tests to transition from epidemiologic tools to clinically actionable diagnostics.

##### **Circulating Tumor HPV DNA (ctHPV-DNA) in Plasma** 

Liquid biopsy approaches represent a paradigm shift in HPV detection, offering real-time, minimally invasive biomarkers that reflect tumor dynamics throughout the disease course.

Liquid biopsy based on circulating tumor HPV DNA (ctHPV-DNA) has rapidly emerged as a powerful non-invasive adjunct to tissue-based testing in HPV-associated OPSCC. Its potential clinical utility spans the entire disease continuum—from diagnosis to treatment monitoring and surveillance—by offering a minimally invasive tool that captures real-time tumor dynamics. However, its performance varies according to detection platform, study design, specimen type, and pre-analytical factors, highlighting the need for critical evaluation before broad clinical adoption.

##### **Diagnostic Performance** 

Meta-analyses have demonstrated the strong diagnostic value of ctHPV-DNA. A 2023 analysis of droplet digital polymerase chain reaction (ddPCR) assays at baseline diagnosis (n = 998) reported pooled sensitivity of 90% (95% CI 82–94%) and specificity of 94% (95% CI 85–98%), when p16/HPV-DNA of tumor biopsy was used as the gold standard [15]. Complementary evidence from a 2024 meta-analysis of TTMV-HPV DNA in surveillance (12 studies, n = 1311) confirmed comparable or superior accuracy, with pooled sensitivity of 86% (78–91%), specificity of 96% (91–99%), and a diagnostic odds ratio > 370 [4]. TTMV-HPV DNA displays fragmentation and epigenetic features characteristic of tumor-derived DNA, distinguishing it from the intact viral DNA typically found in benign oral or genital infections; its measurement in plasma can predict recurrence with high sensitivity and specificity, serving as a valuable complement to imaging and clinical examination. Importantly, ctHPV-DNA positivity often anticipates relapses with a median lead time of ~47 days, although false negatives (~10%) remain a concern, particularly in low-volume disease. Apparent false positives have been reported, more often in young women with unknown cervical HPV status, emphasizing the need for confirmatory testing.

Comparative analyses show that Next-Generation Sequencing (NGS) achieves the highest pooled sensitivity (>95%), surpassing ddPCR and qPCR, while all platforms maintain specificities >95% [15]. Capture-based Next-Generation Sequencing (CaptHPV-NGS) further enables viral genotyping and integration analysis, reaching 95% sensitivity and 98% specificity [15]. Yet, due to cost and technical complexity, ddPCR currently represents the most pragmatic option for routine use. Plasma is preferable to serum, given superior analytical sensitivity, and OPSCC outperforms other HPV-driven cancers in detection accuracy [14].

Most circulating HPV DNA assays have focused on HPV16 detection, reflecting its epidemiological predominance in OPSCC, although multiplex approaches capable of detecting additional high-risk genotypes are increasingly being developed.

##### **Prognostic and Dynamic Monitoring** 

The kinetics of ctHPV-DNA during treatment provide dynamic and clinically meaningful prognostic information in patients with HPV-associated OPSCC. At baseline, the TTMV HPV DNA assay demonstrated 91.5% sensitivity and 100% specificity for detecting active HPV-positive OPSCC when compared with histopathologic and radiologic confirmation as reference standards [30].

During post-treatment surveillance, the same assay showed 88.4% sensitivity and 100% specificity for identifying residual or recurrent disease, validated against imaging and biopsy findings [30]. Importantly, ctHPV-DNA positivity often preceded radiologic or clinical evidence of recurrence by several months, suggesting its potential as an early-warning biomarker for minimal residual disease.

Notably, circulating HPV DNA can also distinguish ‘true’ residual disease, contrasting with the suboptimal positive predictive value of PET-CT. Studies have shown that when ctHPV-DNA is undetectable after treatment, patients with persistent PET-CT uptake frequently show no viable tumor on biopsy, and the radiologic activity subsequently resolves—reflecting the delayed regression typical of HPV-driven tumors. Conversely, residual ctHPV-DNA detected at the end of chemoradiotherapy or during follow-up has been associated with lower disease-free and overall survival, as observed in HPV-related malignancies such as locally advanced cervical cancer [65,66].

Chera et al. [33] further demonstrated that complete ctHPV16-DNA clearance within the first four weeks of chemoradiotherapy was strongly associated with an absence of persistent or recurrent disease, whereas unfavorable clearance kinetics corresponded to a 35% rate of regional recurrence. These data highlight the prognostic relevance of on-treatment molecular response, indicating that ctHPV-DNA kinetics can serve as an early indicator of therapeutic efficacy.

Collectively, these findings emphasize that ctHPV-DNA dynamics capture real-time tumor behavior rather than static disease status. Rapid decline or complete clearance during chemoradiotherapy correlates with improved locoregional control and survival, whereas persistent or rising levels predict recurrence. Accordingly, ctHPV-DNA represents a promising adjunct for adaptive surveillance and potentially for response-guided de-escalation strategies in HPV-associated OPSCC.

Conversely, some studies have reported conflicting findings. For instance, Cao et al. [36] observed that an early rise in ctHPV-DNA two weeks into chemoradiotherapy correlated with improved outcomes, potentially reflecting tumor apoptosis. In contrast, a subsequent trial combining chemoradiation with nivolumab found no such association [38]. These inconsistencies likely stem from methodological variability and treatment heterogeneity, particularly in studies incorporating immunotherapy. Such divergent results underscore the impact of differences in study design—such as sampling timing and assay platform—and highlight the urgent need for standardized protocols in future investigations.

Despite encouraging evidence from prior studies regarding the clinical utility of liquid biopsies, the frequency and timing of blood sample collection have varied considerably, ranging from several weeks to up to two years after treatment. While some studies applied different sampling schedules for different patients, others relied on a single post-treatment blood draw. For example, in the study by Chera et al. [33], the detection of ctHPV-DNA in two consecutive plasma samples during post-treatment surveillance was required to achieve high positive and negative predictive values for recurrence detection. This lack of methodological standardization continues to hinder the translation of these findings into clinical practice.

A low HPV copy number, initially considered a marker of reduced tumor burden, has paradoxically been associated with worse prognosis, likely due to higher degrees of viral integration and oncogenic activation [67]. Thus, longitudinal monitoring of ctHPV-DNA dynamics may serve both as a surrogate of therapeutic response and as a prognostic biomarker to refine risk stratification. The future application of ctHPV DNA involves developing highly sensitive surveillance strategies that can detect disease relapse before it becomes clinically apparent, potentially improving the probability of successful radical salvage therapy [68].

Collectively, the evidence supports ctHPV-DNA as a powerful adjunct for diagnosis, response assessment, and surveillance. However, heterogeneity across platforms and limited clinical standardization still precludes its universal adoption. Ongoing prospective validation will determine whether ctHPV-DNA can evolve from a promising biomarker into a true clinical decision-making tool. Together, these findings demonstrate that ctHPV-DNA is among the most promising biomarkers in head and neck oncology, though methodological harmonization remains a prerequisite for its translation into routine practice.

### 3.4. Clinical Applications

#### 3.4.1. De-Escalation Strategies

There is growing interest in using ctHPV-DNA kinetics to tailor therapy intensity. Prospective phase II trials demonstrate that early favorable clearance may identify patients suitable for treatment de-intensification, while persistence signals the need for full-intensity or even escalated therapy. For example, in a single-center study (NCT04572100, n = 46; 488 samples), rapid clearance (≥95% reduction after one neoadjuvant cycle) correlated with radiographic deep response (*p* = 0.04), whereas detectable ctHPV-DNA ≥ 3 months post-treatment predicted poor progression-free and OS. Trials such as REACT (NCT04900623) and DART 2.0 (NCT05541016) are prospectively evaluating ctDNA dynamics as mid-treatment biomarkers to guide radiotherapy de-intensification. In these phase II trial designs, patients with elevated ctDNA at week 4 continue standard-dose therapy, while those with low levels are allocated to reduced-dose treatment. If there are positive signals from these trials, large, randomized phase III trials will be needed to validate ctHPV-DNA as a tool for risk-adapted, individualized treatment. Of note, a pilot study using TTMV-HPV DNA to select patients for adjuvant RT omission (NCT05307939) did not meet its primary endpoint, with recurrences occurring despite negative ctHPV-DNA, suggesting that ctHPV-DNA may lack sensitivity for detecting minimal residual disease in very early-stage presentations [61].

#### 3.4.2. Surveillance

Besides the study of Chera et al. [33], there are only a few studies with a limited number of patients reporting the value of ctHPV-DNA liquid biopsies to detect recurrent disease during follow-up. Pooling data from these studies [31,32,34,69,70,71] reveals a sensitivity of 94% and a specificity of 99%. Longitudinal monitoring provides high specificity and PPV, with recurrence detectable up to 25 months before clinical or radiographic confirmation [37]. Longitudinal testing with NavDx (TTMV-HPV DNA) has shown sensitivity ~86%, specificity ~96%, PPV up to 100%, and NPV up to 100%. Importantly, the PPV of a single positive test can be as low as 53% but increases to ~94% with two consecutive positives [33].

Given the high sensitivity and negative predictive value of ctHPV-DNA assays, their greatest strength lies in the early detection of recurrence, allowing timely intervention before radiologic or clinical evidence appears. In this context, prioritizing sensitivity is crucial: it is preferable to trigger additional investigations or imaging studies rather than risk missing an early relapse. Although confirmatory testing remains essential to guide management decisions, the potential harm of an unnecessary scan is outweighed by the clinical consequences of a delayed diagnosis.

Large multicenter studies analyzing longitudinal liquid biopsies for HPV using ctHPV-ddPCR and ctHPV-DNA sequencing are currently ongoing in the Netherlands.

#### 3.4.3. SCC of Unknown Primary (SCCUP)

In patients with squamous cell carcinoma of unknown primary (SCCUP), plasma ctHPV16DNA has shown excellent diagnostic performance, with pilot studies reporting 90.9% sensitivity and 100% specificity. While ctHPV16DNA does not directly visualize the tumor, its detection reliably indicates an oropharyngeal origin, enabling accurate discrimination of HPV-driven SCCUP and potentially obviating invasive diagnostic procedures such as TORS [15,40].

#### 3.4.4. Challenges and Future Directions

Despite encouraging results, several challenges still limit the routine implementation of ctHPV-DNA testing. One of the main obstacles is methodological heterogeneity, which operates at multiple levels. First, substantial variability exists across assay platforms, including droplet digital PCR (ddPCR), quantitative PCR, next-generation sequencing (NGS), and tumor tissue-modified viral HPV DNA (TTMV-HPV DNA) approaches, each of which differs in analytical sensitivity, genotype coverage, cost, and scalability. Second, molecular targets are not uniform across studies: some assays focus on HPV16-specific E6/E7 regions, whereas others include broader multi-genotype panels or fragmentomic approaches, making direct comparison difficult. Third, pre-analytical variability remains a major limitation, since specimen type, blood collection tubes, plasma processing time, storage conditions, and DNA extraction methods can all affect assay performance and reproducibility.

Additional heterogeneity arises from the lack of consensus regarding positivity thresholds, reporting units, and the optimal timing and frequency of ctHPV-DNA sampling during treatment and surveillance. These differences complicate interpretation across studies and currently prevent the establishment of universally applicable clinical algorithms. Importantly, many published studies are based on relatively small cohorts, frequently dominated by HPV16-positive OPSCC, which further limits generalizability across clinical settings and HPV genotypes. Large prospective multicenter studies using harmonized protocols will therefore be essential to determine whether ctHPV-DNA-guided management truly improves patient outcomes and cost-effectiveness. Economic and logistical barriers also remain relevant, particularly for NGS-based platforms, which may be difficult to implement routinely in resource-limited settings.

To better illustrate these challenges, Table 2 summarizes the principal sources of heterogeneity in ctHPV-DNA testing, including differences in platforms, molecular targets, and key standardization gaps. Future efforts should focus on harmonizing pre-analytical and analytical procedures, integrating ctHPV-DNA with radiologic and histopathologic data, and establishing rigorous quality control frameworks in order to move this technology from an investigational tool to a validated biomarker suitable for routine clinical practice.

While liquid biopsy offers a biologically direct window into tumor dynamics, computational surrogates represent a complementary frontier—leveraging digital pathology and imaging data to infer HPV status without molecular testing.

#### 3.4.5. Emerging Computational Surrogates for HPV Attribution

Recent years have witnessed the emergence of artificial intelligence and computational pathology tools capable of inferring HPV status from imaging and histopathological data. These technologies may complement or even replace some molecular assays in the future.

##### **Imaging-Based Matching-Learning Prediction** 

Artificial intelligence (AI)–based computational approaches are increasingly explored as complementary tools for HPV detection and risk stratification in OPSCC. Different methodological strategies have been proposed depending on the type of input data. Radiomic approaches have been used to identify the HPV status of OPSCC from CT and MRI imaging, with reported areas under the receiver operating curve (AUC) ranging from 0.70 to 0.80 for CT and from 0.64 to 0.79 for MRI [42,43,44]. These models extract quantitative imaging features associated with tumor morphology and texture that may correlate with HPV-driven tumor biology.

More recently, deep learning architectures has been applied directly to imaging data. A three-dimensional convolutional neural network (3D CNN) fine-tuned from video pre-training has been shown to differentiate HPV-positive from HPV-negative OPSCC using routine diagnostic CT, achieving an AUC of 0.81 in an external test set and outperforming both 3D models trained from scratch and 2D ImageNet pre-training approaches in a cohort of 273 patients. The prognostic relevance of the resulting deep learning-based HPV prediction score (HPV-ps) was subsequently validated in a total of 594 patients. In the same study, the ability of four board-certified pathologists to identify HPV associations from histologic morphology was evaluated and compared with the classifier, highlighting the potential of computational biomarkers as adjunctive tools for risk stratification. Nevertheless, multicenter validation and calibration against transcriptionally active HPV detection remain essential before clinical implementation [45].

In contrast to radiologic approaches, histopathology-based models analyze digitized whole-slide images using patch-based deep learning frameworks or multiple-instance learning strategies that aggregate predictions from multiple tumor regions. In these pipelines, tumor regions may first be identified through segmentation networks such as U-Net, followed by classification architectures such as DenseNet that evaluate multiple tumor patches to generate an overall HPV prediction score. Studies using this approach have reported an AUC of approximately 0.8 for predicting HPV association from routine hematoxylin–eosin–stained slides and have demonstrated potential prognostic stratification when combined with biomarkers such as p16.

Despite these promising results, several challenges remain for clinical translation. Many deep learning models function as “black boxes,” limiting the interpretability of their predictions. Emerging explainable AI techniques, such as gradient-weighted class activation mapping (Grad-CAM), may help identify histologic regions that contribute most strongly to algorithm outputs. In addition, computational models often depend on high-quality digitized slides or standardized imaging protocols. Strategies to improve robustness include training models using multicenter datasets, applying stain normalization and image harmonization techniques, and integrating AI-derived predictions with established molecular biomarkers. At present, these computational approaches should be considered complementary to established HPV diagnostic methods rather than replacements, pending prospective validation in multicenter clinical datasets.

#### 3.4.6. Computational Pathology on H&E Whole Slides

A multiple-instance learning framework with triplet-ranking loss achieved state-of-the-art HPV detection using 431 diagnostic H&E-stained whole-slide images (WSIs) from 412 patients across two HNSCC cohorts, and its scores correlated with microenvironmental features (↑ follicular helper/CD8 T-cells, ↓ macrophages/fibroblasts) and expected gene expression patterns—findings concordant with established HPV-related biology. While promising for screen-triage and research stratification, WSI-based surrogates should currently be considered adjuncts to validated molecular assays [46]. Other platforms have been correlated with outcome yet still require further validation [47].

While not yet ready for standalone clinical use, these computational approaches hold promise as cost-effective triage tools or for providing HPV status in cases where tissue is scarce or degraded. Key challenges to their clinical adoption include the “black box” nature of predictions, dependency on high-quality digitized data, and the risk of poor generalizability to external patient populations and imaging protocols [72].

Although preliminary results are encouraging, computational HPV surrogates remain experimental. Standardization, transparency, and multi-institutional validation will be essential before these models can be safely integrated into diagnostic workflows. The convergence of AI-driven image analysis with molecular diagnostics could redefine HPV attribution workflows, but transparency, interoperability, and clinical validation remain the main barriers to real-world implementation.

## 4. Concluding Remarks and Future Perspectives

Integrating current evidence provides a coherent framework for understanding how HPV detection methods can be applied across diagnosis, prognostication, and therapeutic decision-making. Reliable determination of HPV causality in oropharyngeal squamous cell carcinoma (OPSCC) is fundamental for accurate staging, prognostic assessment, and the safe implementation of treatment de-intensification strategies.

In routine clinical practice, p16(INK4a) immunohistochemistry (IHC) remains the most accessible and highly sensitive frontline surrogate test (≥70% strong nuclear–cytoplasmic staining). However, its limited specificity and variability across anatomical sites and tissue processing demand confirmatory viral testing and support dual-testing strategies. Direct nucleic acid-based assays, such as HPV DNA PCR or in situ hybridization (ISH) and, ideally, E6/E7 mRNA detection by RNA-ISH or RT-PCR, offer the highest diagnostic accuracy by confirming transcriptionally active infection.

**Figure 1:**
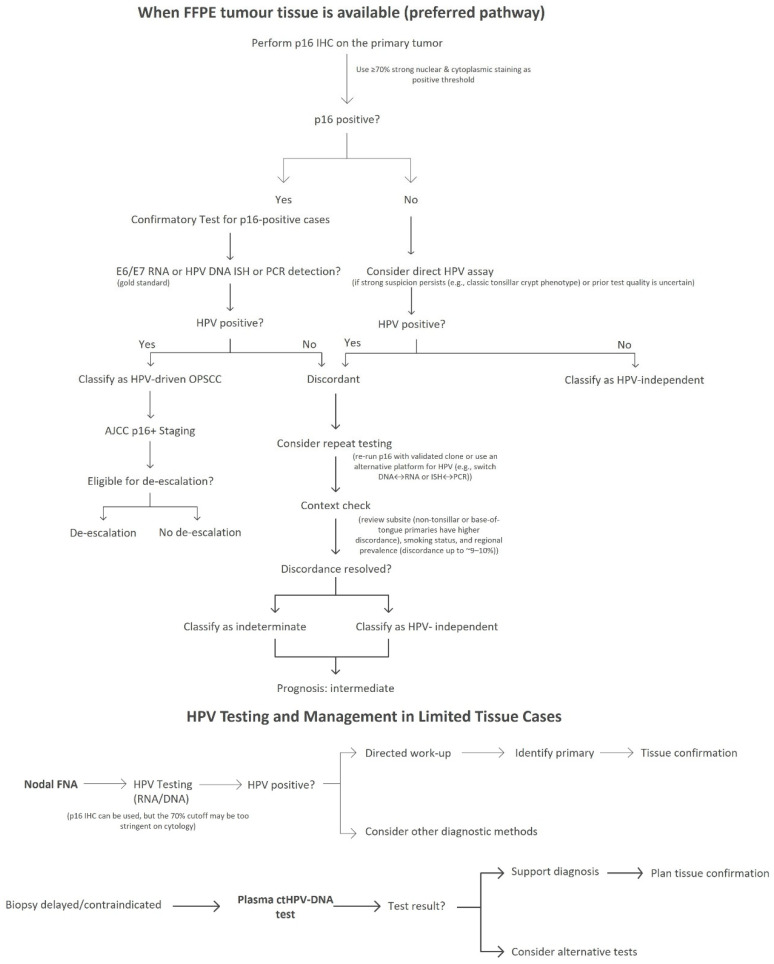
Diagnostic and monitoring algorithm for HPV status in OPSCC. p16 IHC (≥70% strong nuclear/cytoplasmic staining) is the frontline screen; confirmation with a direct HPV assay—preferably E6/E7 RNA (RNA-ISH or RT-PCR), alternatively DNA ISH or DNA PCR—is recommended. Discordant phenotypes (p16+/HPV− or p16−/HPV+) require orthogonal testing and should not be used for de-escalation. When tissue is limited, FNA testing and plasma ctHPV-DNA can bridge to tissue confirmation [14,15,28,49].

Because discordant phenotypes (p16+/HPV− or p16−/HPV+) occur and are associated with intermediate clinical outcomes, diagnostic algorithms emphasize orthogonal testing before any treatment de-escalation. Site-specific caveats, particularly in non-oropharyngeal locations, must also be acknowledged (Figure 1).

**Figure 2:**
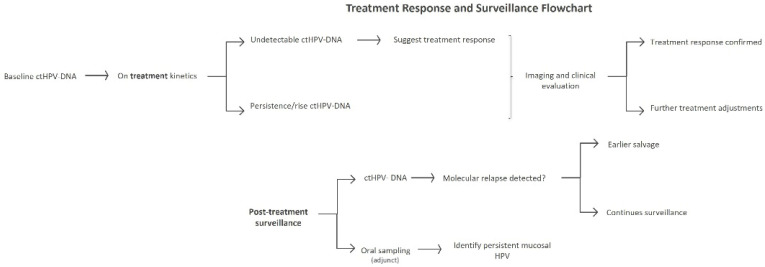
A suggested treatment response and surveillance flowchart [14,15,41].

Beyond tissue analysis, blood-based detection of circulating HPV DNA using ddPCR or NGS has shown high diagnostic accuracy in studied cohorts of HPV-associated OPSCC, with pooled sensitivity and specificity of approximately 90% and 94%, respectively. However, circulating HPV DNA alone cannot definitively determine the anatomic source of HPV DNA, as HPV-derived nucleic acids may originate from non-oropharyngeal HPV-related sites or other HPV-associated conditions. Accordingly, these assays should be interpreted as complementary biomarkers rather than stand-alone diagnostic tools for assigning an oropharyngeal primary. Their clinical value lies in supporting diagnosis, real-time assessment of therapeutic response, and early detection of relapse. More recently, tumor tissue-modified viral HPV DNA (TTMV-HPV DNA) assays have been developed to improve the specificity of circulating HPV DNA detection by focusing on tumor-associated viral DNA fragments. These assays may increase the likelihood that detected HPV DNA originates from tumor tissue; however, they still cannot by themselves definitively establish the anatomic site of origin and should therefore be interpreted within the broader clinical and diagnostic context. By contrast, oral rinse or saliva testing, though practical for identifying oral HPV infection, shows only moderate diagnostic sensitivity and frequently under-samples tonsillar and base-of-tongue disease, as demonstrated by the Oromouth cohort (Figure 2). Consequently, its role remains adjunctive rather than diagnostic for HPV-driven OPSCC.

Although most HPV-positive OPSCC patients experience favorable outcomes, 20–30% still progress or relapse despite standard therapy. Integrating highly sensitive detection methods with comprehensive HPV genotyping—including non-HPV16 subtypes—is critical to refine patient selection for treatment de-intensification and improve prognostic stratification.

In essence, reliable HPV determination requires a multimodal approach: (1) p16 IHC as an initial high-sensitivity screen; (2) direct nucleic acid detection for viral confirmation, and (3) ctHPV-DNA as a complementary biomarker for monitoring and recurrence detection.

As molecular technologies advance, the integration of non-invasive assays, computational tools, and molecular profiling will enable adaptive, precision-oriented diagnostic algorithms. Future efforts must focus on standardizing methodologies and validating integrated diagnostic pathways in prospective trials to fully realize the potential of precision medicine in HPV-mediated OPSCC.

The available evidence remains limited by several factors inherent to the field. Most data derive from retrospective cohorts with heterogeneous testing methodologies, non-standardized cut-offs for p16 positivity, and inconsistent reference standards for viral detection. Few studies directly compare multi-assay algorithms in prospective settings or across diverse healthcare systems, and the representation of non-HPV16 genotypes and non-oropharyngeal sites is still insufficient. Moreover, the clinical utility of emerging biomarkers such as ctHPV-DNA requires validation through multicenter trials with standardized protocols and longitudinal follow-up.

From a clinical perspective, translating these advances into routine practice demands the harmonization of diagnostic workflows and cost-effective implementation in variable laboratory contexts. From a research standpoint, integrating genomic, transcriptomic, and immunologic profiling with artificial intelligence-based analytic frameworks may yield dynamic predictive models capable of refining diagnosis, prognosis, and therapeutic guidance. Ultimately, bridging laboratory precision with real-world applicability will be essential to achieve equitable, evidence-based personalization of care in HPV-driven OPSCC.

As the field evolves, bridging molecular precision with scalable, equitable implementation will determine whether HPV-driven OPSCC becomes not only a biologically distinct disease but also a model for personalized oncology.

## Figures and Tables

**Table 2:** Main sources of heterogeneity and standardization gaps in ctHPV-DNA testing for HPV-associated OPSCC.

Domain	Examples of Heterogeneity	Potential Impact on Results	Current Standardization Gap
**Assay platform**	ddPCR, qPCR, NGS, TTMV-HPV DNA	Different sensitivity, specificity, multiplexing capacity, and cost	No universally accepted platform for diagnosis or surveillance
**Molecular target**	HPV16 E6/E7, multi-genotype panels, viral fragment patterns	Variable genotype coverage and tumor specificity	No consensus on optimal target region(s)
**Genotype coverage**	HPV16-only vs. HPV16/18/31/33/35 or broader panels	Reduced generalizability for non-HPV16 tumors	Limited validation in non-HPV16 OPSCC
**Specimen and pre-analytics**	Plasma vs. serum, collection tubes, processing time, storage conditions, extraction methods	Variation in DNA yield, stability, and reproducibility	No harmonized pre-analytical workflow
**Positivity thresholds**	Absolute copy number, fractional abundance, binary positive/negative definitions	Difficult comparison across studies and risk of inconsistent classification	No standardized reporting thresholds
**Sampling strategy**	Baseline only, serial during treatment, post-treatment surveillance intervals	Different sensitivity for response assessment or recurrence detection	No consensus on optimal timing and frequency
**Clinical setting**	Diagnosis, treatment monitoring, minimal residual disease, surveillance	Performance may vary by indication	No indication-specific testing algorithm
**Validation level**	Single-center retrospective studies vs. prospective multicenter cohorts	Limited external validity and reproducibility	Need for prospective multicenter validation

**ddPCR:** droplet digital polymerase chain reaction; **qPCR:** quantitative polymerase chain reaction; **NGS**: next-generation sequencing; **TTMV-HPV DNA**: tumor tissue-modified viral human papillomavirus DNA; **HPV**: human papillomavirus; **OPSCC**: oropharyngeal squamous cell carcinoma.

## Data Availability

No new data were created or analyzed in this study. Data sharing is not applicable to this article.
